# Experimental Evolution Reveals a Genetic Basis for Membrane-Associated Virus Release

**DOI:** 10.1093/molbev/msaa208

**Published:** 2020-08-18

**Authors:** Juan-Vicente Bou, Rafael Sanjuán

**Affiliations:** Institute for Integrative Systems Biology (I2SysBio), Consejo Superior de Investigaciones Científicas-Universitat de València, Paterna, València, Spain

**Keywords:** viral transmission, directed evolution, ultra-deep sequencing, virus–membrane interactions, enterovirus

## Abstract

Many animal viruses replicate and are released from cells in close association to membranes. However, whether this is a passive process or is controlled by the virus remains poorly understood. Importantly, the genetic basis and evolvability of membrane-associated viral shedding have not been investigated. To address this, we performed a directed evolution experiment using coxsackievirus B3, a model enterovirus, in which we repeatedly selected the free-virion or the fast-sedimenting membrane-associated viral subpopulations. The virus responded to this selection regime by reproducibly fixing a series of mutations that altered the extent of membrane-associated viral shedding, as revealed by full-genome ultra-deep sequencing. Specifically, using site-directed mutagenesis, we showed that substitution N63H in the viral capsid protein VP3 reduced the ratio of membrane-associated to free viral particles by 2 orders of magnitude. These findings open new avenues for understanding the mechanisms and implications of membrane-associated viral transmission.

## Significance

Over the last decade, it has been established that many viruses remodel intracellular membranes to create structures used for viral replication, assembly, and shedding. However, how these virus–membrane interactions are encoded in viral genomes remains unassessed. Here, we have used directed evolution, deep sequencing, and genetic analysis to identify mutations controlling membrane-associated viral shedding in a model enterovirus. This will help elucidate the mechanisms and evolutionary causes of virus–membrane interactions and will allow manipulation of these interactions for basic research and practical applications.

## Introduction

Viruses often disperse as groups of infectious particles instead of as independent virions (Altan-Bonnet 2016; Sanjuán 2017; Mutsafi and Altan-Bonnet 2018; Sanjuán and Thoulouze 2019). A well-documented process leading to the formation of collective infectious units is the association between viral particles and cellular membranes for replication and assembly (Hsu et al. 2010; Belov 2016; Fernández de Castro et al. 2016; Ravindran et al. 2016; Romero-Brey and Bartenschlager 2016; Shulla and Randall 2016; Altan-Bonnet 2017). This can result in the extracellular release of pools of membrane-bound viruses, for instance in the form of virion-containing vesicles, as demonstrated for hepatitis A virus (Feng et al. 2013), enteroviruses (Bird et al. 2014; Robinson et al. 2014; Chen et al. 2015), cardioviruses (van der Grein et al. 2019), noroviruses (Santiana et al. 2018), rotaviruses (Santiana et al. 2018), and marseilleviruses (Arantes et al. 2016).

The fitness advantages and costs of membrane-associated viral shedding and other forms of collective viral spread remain poorly understood. In mice, rotavirus-containing vesicles administered through the gastrointestinal route appeared to be more infectious than free virions (Santiana et al. 2018). This could be due, among other possible reasons, to increased resistance of membrane-bound viruses against antibody-mediated neutralization or certain host proteases. Another important feature of collective infectious units is that they deliver multiple viral genomes copies to the same cell, thereby increasing the cellular multiplicity of infection (MOI). Specifically, it has been shown that coxsackievirus high-weight infectious units made of membrane-bound viruses typically contain tens of viral particles, each capable of initiating a productive infection (Bou et al. 2019). Challenging cells with multiple viral genome copies could accelerate early stages of the infection cycle and allow the virus to better overcome infection barriers (Stiefel et al. 2012; Boulle et al. 2016; Andreu-Moreno and Sanjuán 2018). It has also been suggested that spreading in groups could promote genetic complementation or other positive interactions between different virus variants (Shirogane et al. 2012; Simon et al. 2013; Altan-Bonnet and Chen 2015; Chen et al. 2015; Aguilera et al. 2017; Sanjuán 2017). On the other hand, high MOIs tend to favor the accumulation of defective viruses, thereby reducing mean population fitness (Marriott and Dimmock 2010; Brooke 2017; Manzoni and López 2018; Rezelj et al. 2018). Furthermore, producing collective infectious units entails an inherent cost in terms of dispersal capacity, since a pool of jointly transmitted viral particles infects a single cell, whereas multiple cells could potentially be reached by these same particles if they were transmitted independently (Sanjuán and Thoulouze 2019).

Importantly, the genetic basis underlying membrane-associated viral shedding remains unknown. If this trait was under the genetic control of the virus, it should be optimizable by natural selection. Alternatively, this process could be entirely determined by the cell, the virus playing a purely passive role. Here, we use experimental evolution to investigate the genetic basis and fitness implications of membrane-associated shedding in coxsackievirus B3 (CVB3), a model enterovirus. Enteroviruses are released from cells both as free virions and as pools of membrane-associated particles, typically phosphatidylserine-rich vesicles containing multiple virions (Bird et al. 2014; Robinson et al. 2014; Chen et al. 2015). By repeatedly selecting one or the other subpopulation, we identified mutations altering the ability of CVB3 to be transmitted collectively in a membrane-associated manner. Specifically, we found that substitution N63H in the viral capsid protein VP3 strongly reduced membrane-associated viral shedding. This mutation increased viral fitness under our experimental conditions, suggesting a cost for this type of collective spread in CVB3.

## Results

### Directed Evolution of Free versus Membrane-Associated CVB3 Spread

Enterovirus-infected cells release a mixture of free virions and pools of membrane-associated viral particles (MAVPs). These two subpopulations can be separated by low-speed centrifugation on the basis of their different size and weight (Chen et al. 2015). To achieve this, we inoculated HeLa-H1 cells with CVB3 (Nancy strain), harvested the culture medium at 20 h post inoculation (20 hpi), and centrifuged this medium at 10,000 × g. To better separate free viral particles from MAVPs, we performed three serial centrifugation/resuspension rounds, as shown previously (Bou et al. 2019). The supernatant of the first centrifugation constituted our free-virion-enriched fraction (S), whereas the resuspended pellet of the third centrifugation round constituted our MAVP-enriched fraction (P). Plaque assays indicated that the S fraction contained on average (1.8 ± 0.1) × 10^8^ plaque forming units (PFU) per milliliter, whereas the titer of the P fraction was (1.1 ± 0.3) × 10^7^ PFU/ml.

**Fig. 1.**
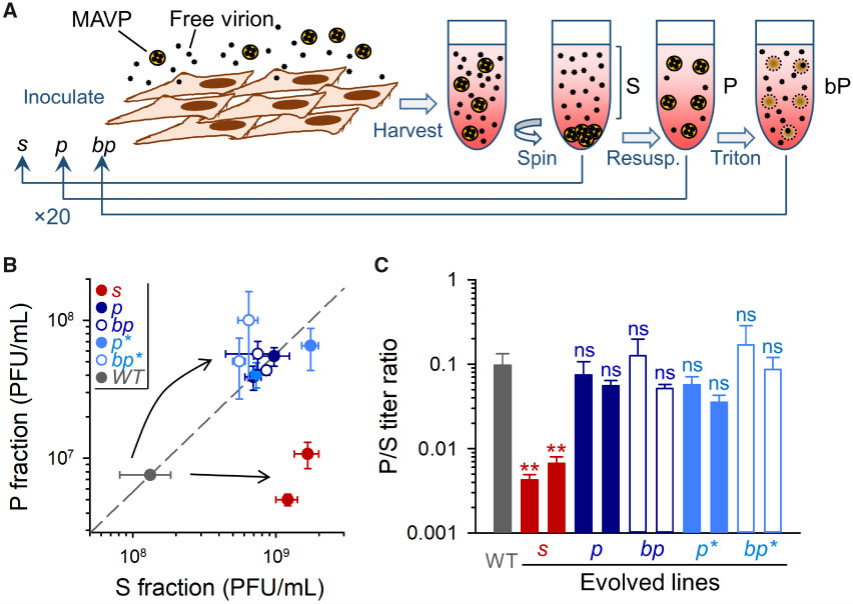
Directed evolution of membrane-associated viral shedding. (*A*) Scheme of the evolution protocol. Free virions and fast-sedimenting MAVPs were separated by low-speed centrifugation. S (supernatant) or P (pellet) fractions were used for inoculating fresh cells, and this process was iterated 20 times. A different P selection regime consisted of treating P fractions with Triton X-100 to disrupt membranes and release free particles (*bp* lines). Furthermore, we explored a variant of the MAVP selection regime by using annexin V for purifying phosphatidylserine-rich particles from P fractions (p* and bp* lines, not shown). (*B*) Titers of the S and P fractions for the WT founder and each of the evolved lines. The dashed line indicates the P/S titer ratio of the founder. (*C*) P/S titer ratio for the founder virus and evolved lines. Error bars correspond to the standard error of the mean (SEM, *n *=* *3 replicates). Asterisks indicate the statistical significance of a log-scale *t*-test against the founder (ns, nonsignificant; **P <* *0.01).

To promote the evolution of virus variants with different abilities to propagate as pools of membrane-bound particles, we performed 20 serial transfers in which we inoculated cultures with 0.1 PFU per cell, harvested the infection media at 20 hpi, and systematically selected the S or P fraction for the following transfer (fig. 1*A*). We will refer to these evolution lines as *s* and *p*, respectively. Two replicate evolution lines of each type were performed (*s1*, *s2*, *p1*, and *p2*). As an alternative selection regime, we performed two additional evolution lines in which the P fraction was purified after each transfer with magnetic beads conjugated to annexin V (P* treatment). This will be referred to as p* lines. Annexin V is a protein that selectively binds phosphatidylserine, a lipid found abundantly in enterovirus–membrane complexes (Chen et al. 2015). Finally, we performed two additional replicate evolution lines (called *bp*) in which the P fraction was treated with a detergent (Triton X-100) after each transfer to disrupt membranes and release free viral particles, and two more lines in which the same procedure was applied to P* fractions (bp* lines). Since MAVPs constitute collective infectious units (Chen et al. 2015; Bou et al. 2019), we reasoned that use of native P fractions could increase the cellular MOI at inoculation, and that this might reduce the efficiency of selection by allowing the accumulation of nonadaptive mutations through genetic complementation. By disrupting MAVPs in P fractions and inoculating cells at 0.1 PFU/cell, we ensured that each cell received no more than one infectious particle on average. In total, thus, we performed five different selection regimes (*s*, *p*, p*, *bp*, and bp*), with two replicate evolution lines per treatment (total: ten lines).

To assess the response to selection after the 20 transfers, we carried out the low-speed centrifugation P/S separation protocol for the wild-type (WT) founder and the evolved lines in the same experimental block, and titrated each fraction. All evolved lines showed overall titers ∼1 order of magnitude higher than the WT founder, suggesting adaptation to cell culture conditions. However, the titers of the S and P fractions varied differently depending on the line (fig. 1*B*). In *p*, p*, *bp*, and bp* lines, the titers of the S and P fractions increased by a similar amount, such that the P/S titer ratio remained approximately similar to that of the WT (fig. 1*C*). In contrast, in *s* lines, only the titer of the S subpopulation increased and, consequently, P/S ratios decreased by an order of magnitude (0.430 ± 0.063% and 0.667 ± 0.137% for *s* lines, vs. 9.75 ± 3.5% for the WT; *t*-tests of log P/S against WT: *P *<* *0.01; fig. 1*C*). Hence, *s* lines responded to selection as expected, whereas lines in which we favored MAVPs (*p*, p*, *bp*, and bp*) did not evolve higher P/S ratios.

### Possible Fitness Costs of MAVP Production

**Fig. 2.**
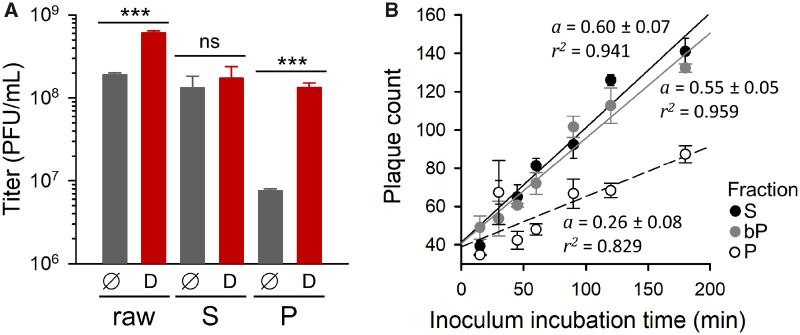
Potential fitness costs of membrane-associated viral shedding. (*A*) Effect of Triton X-100 treatment on the titer of raw infection media, S and P fractions for the WT founder (D: detergent; ∅: untreated controls). Error bars correspond to the SEM (*n *=* *3 replicates). Asterisks indicate the statistical significance of a log-scale *t*-test against the founder (ns, nonsignificant; ***P <* *0.001). (*B*) Increase in the number of PFUs as a function of inoculum incubation time in standard plaque assays using S-fraction, P-fraction, and bP inocula. Regression lines are shown (*a*: slope; *r*^2^: coefficient of determination). Error bars correspond to the SEM (*n *=* *3 replicates).

A simple interpretation of the fact that we successfully promoted the evolution of reduced P/S ratios but we did not obtain increased P/S ratios is that membrane-associated viral spread incurred a fitness cost under our experimental conditions. As discussed above, there are several possible reasons for this. The accumulation of maladaptive mutations through genetic complementation was unlikely, since *bp* and bp* lines appeared to evolve similarly to *p* and p* lines. Yet, a fitness cost could originate from the lower number of PFUs formed by MAVP pools compared with equal numbers of free viral particles. To test this, we used Triton X-100 for disrupting membranes from a WT-infected culture. Detergent treatment of the raw infection medium (no S/P separation) increased titer by 3.2 ± 0.4-fold, revealing the presence of MAVP pools. The effect of detergent treatment was highly exacerbated in P fractions, which experienced a titer increase of 18.0 ± 2.9-fold. In contrast, this treatment increased the titer of the S fraction only by 1.3 ± 0.1-fold (fig. 2*A*). These results reveal a cost associated with MAVP shedding in terms of the number of infectious units formed by a given amount of progeny viral particles, which should in turn have an impact on the dispersal capacity of the virus.

Another possible cost of producing MAVPs is that particles inside membranous structures could have reduced access to the CVB3 receptor, making virus binding or entry less efficient than for free viral particles. To test this, we measured the rate at which WT P or S fractions adsorbed to cells in a standard plaque assay. The number of PFUs increased with inoculation time approximately twice as fast for S-fraction viruses as for P-fraction viruses (fig. 2*B*). In contrast, when P fractions were treated with Triton prior to inoculation, their adsorption rate became very similar to that of the S fraction, indicating that virus–membrane associations were probably responsible for the lower adsorption efficiency of P-fraction viruses. We therefore conclude that MAVP production probably incurred at least two types of fitness costs, namely lower dispersal capacity and reduced cell binding or entry rates. This may contribute to explaining why our *p*, p*, *bp*, and bp*selection regimes did not efficiently promote the evolution of increased P/S ratios.

### Genetic Analysis of the Evolved Lines

To ascertain the genetic changes arisen during the directed evolution process, the WT founder and transfer-20 viruses were used for RNA extraction, reverse transcription polymerase chain reaction (RT-PCR), and full-length ultra-deep Illumina MiSeq sequencing. This revealed abundant parallel molecular evolution (identical sequence changes in different lines; table 1). Amino acid replacement N63H in the VP3 protein reached frequencies >95% in both *s* lines, whereas it was essentially absent (<2%) from all other lines as well as the founder. Substitution N18K in the VP4 protein was also found specifically in *s* lines, albeit at lower frequencies. On the other hand, residue K257 in the VP1 protein was substituted for Q or M in >99.0% of the reads from the four *p* and p* lines, whereas >99% of the sequences from both *s* lines and the founder contained K. In the four *bp* and bp* lines, the 257M variant also reached high frequencies, but the Q variant was absent. Finally, a I24T polymorphism in the VP4 protein was found in MAVP-selection lines (*p*, p*, *bp*, and bp*), whereas this variation was largely absent from *s* lines and the founder.

**Table 1.** Sequence Analysis of the Evolved Lines.^a^

Mutation	Protein	Residue Change	*s1*	*s2*	*p1*	*p2*	*bp1*	*bp2*	p*1	p*2	bp*1	bp*2
G749A	VP4	A3T	54.4									
U796A		N18K	*8.9*	*63.8*								
U808A		N22K								6.7		
U813C		I24T			*77.6*	*57.1*	*64.5*	*44.7*	*27.4*	*33.3*	*55.7*	*46.6*
U992A	VP2	S15T	5.6	10.7								
G1577A		V210I	8.1									
G1622A		V225I	5.7	20.0								
G1842U	VP3	R35M				5.9						
A1925C		N63H	*98.9*	*97.7*								
U2175C		V146A				6.1			16.0			
A2690G	VP1	K80E			10.8					18.7		
U2696A		S82T	20.0									
G2900A		V150I	19.9	30.1			12.9					
A3005G		I185V				8.8			28.7			
A3140G		K230E								24.6		
A3221C		K257Q^b^			*80.6*	*72.8*			*42.5*	*49.8*		
A3222U		K257M^b^			*18.9*	*26.5*	*84.5*	*96.7*	*56.9*	*49.4*	*99.3*	*98.2*
A3231G		N260S								6.0		
U3302C	2A	F3L						7.2				
U3303C		F3S	57.6									
C3519U		S75L		28.8								
G3810A	2B	C22Y		17.0			6.5					
A3822G		N26S			5.4							
A5093G	3A	I22V			7.9							5.3
C5198U		H57Y	20.4									
G5205C		S59T					7.9					
G5312A	3B	V6M					7.6					
U5952A	3D	F14Y		18.7								
U6405C		I165T							11.3			9.9
A6905C		I332L								19.6		
A7019G		T370A		6.5								

a The observed population frequency (%) of each mutation is shown only for nonsynonymous substitutions present at >5% frequency. The founder virus population contained no such mutations and hence is not shown. A more exhaustive list of mutations is provided in supplementary table S1, Supplementary Material online. Mutations showing >50% frequency in at least one line and present at >5% frequency in at least one additional line are shown in italics. These were selected for further analysis.

b Sequences contained the A3221C or A3222U substitution, but never both together.

**Fig. 3.**
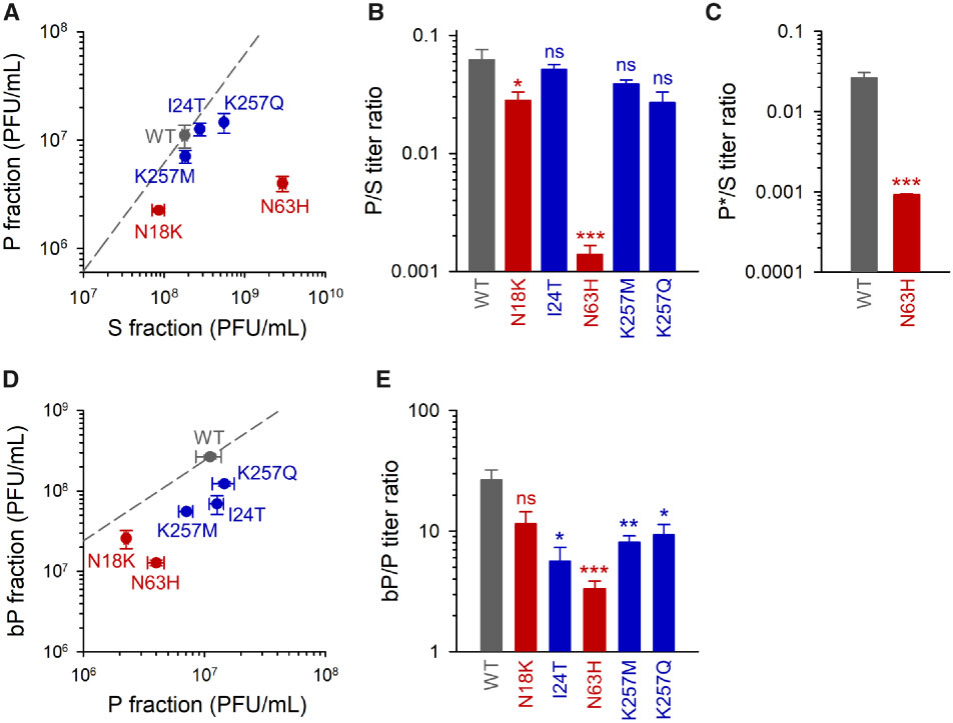
Genetic analysis of the evolved lines. The indicated mutations were introduced in a WT infectious cDNA clone. Mutations that emerged in *s* lines are shown in red and those evolved in *p*, *bp*, p*, or bp* lines are shown in blue. (*A*) Titers of the P and S fractions. The dashed line indicates the P/S titer ratio for the WT. (*B*) P/S titer ratio for the WT and each of the mutants. (*C*) P*/S titer ratio for the WT and N63H mutant. (*D*) Titer of the P fraction and of the P fraction after Triton X-100 treatment (bP). The dashed line indicates the bP/P titer ratio for the WT. (*E*) bP/P titer ratio for the WT and each of the mutants. Error bars correspond to the SEM (*n *=* *3 replicates). Asterisks indicate the statistical significance of a log-scale *t*-test against the founder (ns, nonsignificant; *P <* *0.05, **P <* *0.01, and ***P <* *0.001).

We selected the above five substitutions (N63H, K257Q, K257M, N18K, and I24T) for site-directed mutagenesis analysis. A CVB3 infectious clone identical to the WT founder was used for introducing each of these substitutions individually. For each mutant, we carried out the P/S separation protocol as above to test for changes in the production of MAVPs (fig. 3*A* and *B*). None of the mutations that emerged in MAVP-selection lines (I24T, K257M, and K257Q) had a significant effect on the P/S titer ratio. In contrast, *s*-line mutations reduced the P/S ratio significantly. Whereas the effect of N18K was modest (P/S = 2.81 ± 0.50%, vs. 6.22 ± 1.37% for the WT, *t*-test: *P *=* *0.042), the N63H mutation produced a 40-fold drop in the P/S ratio (0.139 ± 0.027%; *t*-test: *P *<* *0.001), elevating the S titer by 16-fold (*t*-test: *P *<* *0.001) and reducing the P titer by 2.8-fold (*P *=* *0.025). This suggests that N63H increases the production of free viral particles and reduces shedding of fast-sedimenting MAVPs.

To further test whether the N63H mutation modified the ability of the virus to spread in a membrane-associated manner, we used annexin V magnetic beads to pull phosphatidylserine-rich infectious units (P* fraction). We found that the P*/S titer ratio was 30-fold lower for the N63H than for the WT (0.092 ± 0.003% vs. 2.63 ± 0.43%; *t*-test: *P *<* *0.001; fig. 3*C*). Specifically, the P* titer of the N63H mutant was (1.74 ± 0.17) × 10^6^ PFU/ml, versus (2.08 ± 0.32) × 10^7^ PFU/ml for the WT, which represents a 12-fold reduction in the number of annexin-bound PFUs (*t*-test: *P *<* *0.001). This again revealed that the N63H mutation reduced MAVP production.

To determine how the above mutations changed the size of MAVP pools, we subjected P fractions to Triton X-100 treatment to break membrane–virus associations and measured the fold change in titer (bP/P ratio). The increase in titer produced by the detergent treatment was significantly less marked for mutants N63H, I24T, K257M, and K257Q than for the WT (*t*-tests: *P *<* *0.05). The bP/P ratio was lowest for N63H (3.34 ± 0.52 vs. 26.6 ± 5.7 for the WT; *P *=* *0.001; fig. 3*D* and *E*), suggesting that N63H reduced both the number of fast-sedimenting PFUs and the number of infectious particles in these PFUs. Overall, the ratio of MAVPs to free infectious particles (bP/S ratio) was 300-fold lower for the N63H mutant (0.0045 ± 0.0007) than for the WT (1.50 ± 0.07; *P *<* *0.001). For I24T, K257M, and K257Q mutants, albeit similar numbers of fast-sedimenting PFUs were produced, these appeared to contain fewer infectious particles each, as suggested by significantly lower bP/P ratios (fig. 3*D* and *E*).

Since the VP1 K257, and VP4 I24T replacements necessarily occurred in the same viral genomes, we also constructed the double mutants I24T + K257Q (DM1) and I24T + K257M (DM2) and determined their P and S titers. DM1 reduced the P/S ratio similarly to K257Q (1.91 ± 0.15%; *t*-test against WT: *P *=* *0.016), whereas DM2 had little or no effect on P/S ratio (6.23 ± 0.20%; *P *>* *0.05). Overall, both mutants showed properties similar to their respective single mutants, thus revealing no obvious interactions between the I24 and K256 sites.

### Effects of VP3 Mutation N63H on Viral Spread and Fitness

**Fig. 4.**
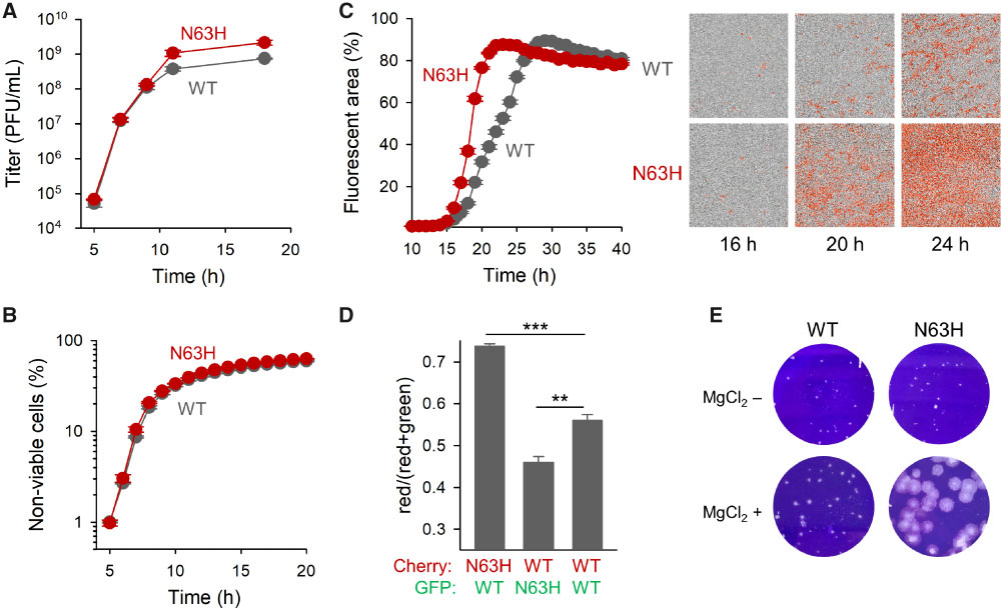
Effects of the N63H mutation on viral titer, spread, and fitness. (*A*) One-step growth curves initiated with 10 PFU/cell of S-fraction virus. (*B*) Cytotoxicity curve (10 PFU/cell of S fraction). (*C*) Viral spread in cultures inoculated at 0.001 PFU/cell, quantified by automated real-time fluorescence microscopy using CVB3-mCherry viruses (left: fluorescence-based growth curve; right: representative images taken at 4× magnification in the phase contrast and red channels). (*D*) Head-to-head competition assays between N63H and WT using eGFP and mCherry reporters (1:1 input ratio, 0.001 PFU/cell at inoculation). The red to total fluorescent areas at 20 hpi are shown for each competition. Error bars correspond to the SEM (*n *=* *3 replicates). Asterisks indicate the statistical significance of a log-scale *t*-test against the founder (**P <* *0.01 and ***P <* *0.001). (*E*) Plaques in agar semisolidified medium with or without MgCl_2_.

To further characterize the N63H mutant, we performed one-step growth curves by inoculating cultures with 10 PFUs per cell. Initially, the N63H and WT variants grew similarly fast but, in later stages, N63H released more PFUs than the WT (fig. 4*A*). The N63H mutation thus increased the number of progeny PFUs released per cell but did not accelerate early progeny production. We also analyzed the cytotoxicity of each variant using a fluorescent marker to quantify membrane integrity loss in cultures inoculated with 10 PFU/cell. We found very similar cell death dynamics for WT and N63H viruses (fig. 4*B*).

The observation that the N63H virus produced more PFUs per cell than the WT could be attributed to the fact that most of the N63H progeny was released as free viral particles instead of as MAVPs. This should allow the N63H progeny to reach more cells, accelerating viral spread compared with the WT. To test this, we used WT and N63H viruses carrying the mCherry reporter. We inoculated cell cultures with a low virus dose (0.001 PFU/cell) and followed viral spread by automated real-time quantitative fluorescence microscopy (fig. 4*C*). As expected, N63H spread faster throughout the cell population than the WT. At 20 hpi, the N63H mCherry signal reached 76.5 ± 0.6% of the cells, versus 31.7 ± 0.9% for the WT (*t*-test: *P *<* *0.001).

Based on this, we expected that N63H should outcompete the WT in head-to-head competition assays. To verify this, we used WT and N63H viruses encoding mCherry or eGFP reporters. When we infected cultures with a 1:1 mixture of N63H-mCherry and WT-eGFP (0.001 PFU/cell), the percentage of mCherry-positive to total fluorescent cells increased to 73.7 ± 0.6% at 20 hpi, whereas in control assays performed with WT-mCherry and WT-eGFP viruses this percentage was 55.9 ± 1.4% (*t*-test: *P *<* *0.001; fig. 4*D*). In contrast, when the infection was initiated with a 1:1 mixture of N63H-eGFP and WT-mCherry, the percentage of mCherry-positive to total fluorescent cells was 45.9 ± 1.5% at 20 hpi (*t*-test against WT–WT competition: *P *=* *0.007). Hence, as expected, the N63H mutant was fitter than the WT in direct competition assays.

The more efficient diffusion of the N63H virus was also shown by measuring plaque sizes in agar-solidified medium. Average N63H plaque size at 44 hpi was ∼2-fold higher (0.0070 ± 0.0007 mm^2^) than that of the WT (0.0032 ± 0.0003 mm^2^; *t*-test: *P *<* *0.001). This difference was highly exacerbated when we added MgCl_2_ to the agar-solidified medium (20-fold larger plaques; 0.136 ± 0.010 mm^2^ vs. 0.0045 ± 0.0004 mm^2^, respectively; *P *<* *0.001; fig. 4*E*). Previous work showed that binding to sulfated glycans hampers the diffusion of CVB3 virions in agar, and that VP3 replacement N63Y reduces virion affinity for sulfated glycans, enlarging plaque size strongly (Wang and Pfeiffer 2016). It is thus possible that N63H also reduces virion affinity for sulfated glycans, but that this effect is MgCl_2_ dependent. Alternatively, both WT and N63H virions might show reduced affinity for sulfated glycans in the presence of MgCl_2_ but, by producing fewer MAVPs and more free particles, the N63H virus would diffuse more efficiently than the WT.

## Discussion

Our results reveal that membrane-associated viral shedding has a genetic basis in CVB3. We found five amino acid substitutions that reached high frequencies in at least two independent evolution lines and that were specific to *s* lines or to some or all MAVP-selection lines (*p*, p*, *bp*, and bp*). Within the latter group, the 257M variant was favored over the 257Q variant in the four *bp* and bp* lines, whereas it was similarly or less abundant than the Q variant in the four *p* and p* lines. It is possible that the M variant was fitter than the Q variant but incurred some fitness cost related to between-transfer transmission. As we have shown, this cost could be related to the fact that MAVPs exhibited less efficient adsorption than free particles. By disaggregating membranes before each transfer, we might have alleviated this cost and favored the 257M variant over 257Q.

The N63H substitution in the VP3 protein had the clearest effect on MAVP shedding. N63H increased the number of PFUs that remained in the supernatant after low-speed centrifugation and reduced the number of PFUs in pellets. N63H infectious units also showed reduced affinity for annexin V. Additionally, N63H diminished the effect of detergent-induced membrane disruption on viral titer. Overall, N63H reduced the proportion of viral progeny shed as MAVPs by ∼300-fold. As expected from the presumable dispersal cost of collective infectious units, N63H improved viral fitness by accelerating viral spread in cell cultures. In contrast, N63H did not accelerate the cellular infection cycle and did not increase viral cytotoxicity.

Residue N63 is located at the virion surface, near the binding site of the decay-accelerating factor, which functions as an alternate cellular receptor for CVB3 when the coxsackievirus adenovirus receptor is not accessible (Yoder et al. 2012). The N63Y mutant was reported in CVB3-infected mice (Wang and Pfeiffer 2016). Furthermore, N63Y, N63S, N63D, and N63H evolved in viruses serially transferred in HeLa cells or A549 lung cells (Bordería et al. 2015). Since in HeLa cells both decay-accelerating factor and coxsackievirus adenovirus receptor are available at the cell surface, it is possible that these N63 mutations were not responsible for changes in receptor usage. As shown previously (Wang and Pfeiffer 2016), residue N63 appears to determine certain virion surface properties. Based on this and on our own results, we suggest that N63 is involved in virus–membrane interactions. However, additional functions for this residue cannot be ruled out.

The fitness advantage of mutation N63H appears to reside in a higher PFU yield per cell, as determined by the higher number of progeny viruses released as free viral particles compared with MAVPs. For loss of membrane-associated viral shedding to change fitness appreciably, a sufficiently large portion of WT progeny should be in the form of MAVPs. Our data suggest that this is the case. Let us assume that the total titer in raw media is T=v+m, where *v* is the number of free-virion PFUs and *m* the number of PFUs constituted by MAVP pools. The overall titer following detergent treatment should be T′=v+nm, where *n* is the average number infectious particles harbored in MAVP pools. The proportion of MAVPs to total infectious particles should thus be f=nm/(v+nm), which can be calculated as f=n/(n-1)(1-T/T′). Since detergent treatment of raw culture media increased WT titer by 3.2-fold, we estimate T′/T= 3.2. The fold increase in titer produced by detergent treatment of the P fraction (bP/P) provides an estimate of *n*. Using *n *=* *26.6, we obtain *f *=* *0.71. Assuming larger *n*-values would produce essentially no change in *f*, since $\lim_{n\to \infty }\left(f\right)=\;(1-T/T\prime )=$ 0.69, whereas lower *n*-values would lead to higher estimates of *f*, up to *f *=* *1 for *n *=* *3.2 (supplementary fig. S1, Supplementary Material online).

The above calculations indicate that a large proportion of the WT progeny is actually released in the form of membrane-bound particle pools. Our systematic selection of S fractions successfully reduced MAVP production, as evidenced by the emergence of the N63H mutant. In contrast, selection of P fractions had a less obvious effect. As we have discussed, a simple explanation for these results is that MAVP production incurs certain fitness costs. Despite a lack of response to selection in terms of P/S ratios, *p*, p*, *bp*, and bp* lines did evolve several parallel mutations that were absent from *s* lines, indicating a specific yet unexpected response to selection. Some of these mutations reduced bP/P ratios, suggesting the production of pools of membrane-bound particles containing fewer viral particles each. We speculate that this might have allowed these lines to produce MAVPs with reduced dispersal costs. Alternatively, our protocols might have inadequately selected MAVPs, but we find this unlikely since we previously showed that P and P* fractions are highly enriched in MAVPs compared with raw cultures (Bou et al. 2019). Considering that this was the first study addressing the evolution of MAVP shedding, we had no previous information on which type of selection regime should work best. We hence explored different selection strategies, which inevitably came at the cost of reducing the number of evolution replicates per treatment. Future work might reveal additional mutations affecting this trait both by exploring new selection regimes and by increasing the number of evolution replicates.

In contrast to the cell culture conditions used here, membrane-associated viral shedding might afford the virus certain fitness advantages in vivo, such as increased infectivity in relatively nonpermissive cell types or resistance to circulating antibodies or proteases. The N63H mutant should provide a useful tool for elucidating the mechanistic basis and in vivo fitness effects of membrane-associated viral transmission.

## Materials and Methods

### Cells and Virus

HeLa-H1 cells were obtained from the American Type Culture Collection (ATCC, CRL-1958) and cultured in Dulbecco’s modified Eagle’s medium (DMEM) supplemented with 10% fetal bovine serum (FBS), nonessential amino acids, 10 units/ml penicillin, 10 µg/ml streptomycin, and 250 ng/ml amphotericin B at 37 °C and 5% CO_2_. Cells tested negative for mycoplasma by PCR. The CVB3 Nancy infectious clone was obtained from Dr Marco Vignuzzi (Pasteur Institute of Paris, France). The mCherry- and eGFP-expressing CVB3 infectious clones were described in our previous work (Bou et al. 2019).

### Virus Titration by the Plaque Assay

Cells in six-well plates were inoculated with 200 µl of virus supernatant in DMEM for 45 min or the indicated time for adsorption assays, the inoculum was removed by aspiration, and cells were overlaid with 2 ml of culture medium containing 2% FBS and supplemented with 0.8% noble agar. At 44–48 hpi cells were fixed by adding 2 ml of 10% formaldehyde to each well for 1 h. Cells were stained with 2% crystal violet in 10% formaldehyde and PFUs were counted.

### Plaque Size Measurement

Plaque assays were performed as indicated and pictures were taken in a Leica MZ10 F microscope at 44 hpi. Pictures were analyzed with Fiji (ImageJ) for plaque size measurement.

### Directed Evolution

Confluent monolayers in six-well dishes were inoculated with 200 µl of virus in DMEM (0.1 PFU/cell) and incubated at 37 °C for 45 min. The inoculum was removed by aspiration after 45 min, and DMEM containing 2% FBS media was added without further washing. After 20 h of incubation at 37 °C, the culture medium was collected without freeze–thawing to avoid vesicle disruption. The relevant fraction (S, P, P*, bP, or bP*) was selected as indicated below, diluted conveniently, and used for the following transfer. Lines were propagated in this way for 20 transfers. In each passage, selected fractions were titrated by the plaque assay. Since passages were done daily (except during weekends) but plaque assays required 44- to 48-h incubations, titers and actual PFU/cell ratios at inoculation were evaluated retrospectively. No major deviations from the desired PFU/cell ratios were found. Specifically, the geometric mean PFU/cell ratio at inoculation ranged between 0.108 and 0.188 for the ten evolution lines across transfers.

### Selection of S Fractions

The culture medium was centrifuged at 1,000 × g for 10 min at 4 °C to remove large cellular debris. The pellet was discarded and the supernatant was centrifuged at 10,000 × g for 10 min at 4 °C to pellet fast-sedimenting PFUs. The supernatant of this centrifugation was collected, stored at 4 or –70 °C, and diluted conveniently to inoculate cells in the following transfer.

### Selection of P Fractions

The culture medium was centrifuged at 1,000 × g for 10 min at 4 °C to remove large cellular debris. The pellet was discarded and the supernatant was centrifuged at 10,000 × g for 10 min at 4 °C to pellet fast-sedimenting PFUs. The pellet was resuspended and centrifuged twice more in the same manner to further wash out free virions. The third pellet was resuspended, stored at 4 °C, diluted conveniently, and used for the following transfer. P fractions were maintained no more than 3 h at 4 °C before use except for the weekends, during which P fractions were stored for 48 h. To verify that MAVPs were stable, we performed a pilot experiment in which the P fraction was stored at 4 °C for 3 weeks. Fresh and 3-week-old P fractions were then compared by subjecting them to detergent-based membrane disruption and obtaining the bP/P titer ratios as an indicator of MAVP integrity. The bP/P titer ratio dropped by only 1.5-fold after 3 weeks.

### Selection of P* Fractions

To perform annexin-based enrichment of MAVPs, P fractions were resuspended in 80 µl of binding buffer provided in an Annexin V microbeads kit (Milteny Biotec, CA) and 20 µl of microbeads were added, incubated for 15 min at 4 °C, and passed through MS columns (Milteny Biotec) following manufacturer’s instructions. These preparations were stored at 4 °C and diluted conveniently for inoculating the following transfer.

### Selection of bP Fractions

To achieve detergent-based membrane disruption, P fractions were incubated in 0.16% Triton X-100 for 15 min at 4 °C, diluted conveniently, and used immediately for the following transfer.

### Response to Selection

To evaluate the effect of the directed evolution protocol on the selected trait or the effect of engineered mutations, confluent monolayer in six-well dishes was inoculated with 200 µl of virus in DMEM (0.1 PFU/cell) and incubated at 37 °C for 45 min, the inoculum was removed by aspiration after 45 min, DMEM containing 2% FBS media was added, and the supernatant was collected at 20 hpi. To quantify the P/S ratio, we performed the P selection protocol detailed above. Specifically, the supernatant obtained after the first centrifugation at 10,000 × g for 10 min at 4 °C was saved as the S fraction, and the protocol was completed to obtain the P fraction. These two fractions were then titrated in parallel. To quantify the effect of detergent-based MAVP disruption on titer, P fractions were incubated in 0.16% Triton X-100 for 15 min at 4 °C and assayed by the plaque assay in parallel with untreated controls. All assays were performed in triplicate.

### RNA Extraction, Amplification, and Deep Sequencing

RNA was obtained from 100 µl of the raw infection media of each evolved line and the founder using the Quick-RNA Viral Kit (ZymoResearch). RNA was reverse transcribed with AccuScript Hi-Fi Reverse Transcriptase (Agilent) using the following primer: 5′TTTTTTTTTTTTTTCCGCAC. The whole viral genome (except primer-annealing regions in 3′ and 5′genome ends) was amplified in three PCR products with Phusion High-Fidelity DNA Polymerase (Thermo Scientific) under the following thermal profile: an initial denaturation at 98 °C for 1 min, 35 cycles of 98 °C for 10 s, 20 s at the indicated annealing temperature, and 72 °C for the indicated time, followed by 5 min of final extension at 72 °C using the following pairs of primers: CV-1F (5′TTAAAACAGCCTGTGGGTTGA) and CV-2143-R (5′GGCCGAACCACAGAACATAA) with an annealing temperature of 64 °C and an extension time of 75 s; CV-2045-F (5′TCGAGTGTTTTTAGTCGGACG) and CV-4923-R (5′AGCCTTCCCACACACAAGAGG) with an annealing temperature of 63 °C and an extension time of 100 s; and CV-4879-F (5′AACATGCCCATGTCAGTCAAGAC) and CV-7399-R (5′CGCACCGAATGCGGAGAA) with an annealing temperature of 66 °C and an extension time of 90 s. PCR products were purified with the DNA Clean & Concentrator kit (ZymoResearch) and quantified by spectrometry (NanoDrop One, Thermo Scientific) to prepare an equimolar mix of the amplicons for paired-end MiSeq Illumina sequencing.

### Analysis of Sequence Data

The quality of the reads was evaluated with FastQC 0.11.7 (www.bioinformatics.babraham.ac.uk/projects/fastqc, last accessed February 11, 2019), trimmed with Cutadapt (cutadapt.readthedocs.io/en/stable/, last accessed February 11, 2019) to clip the first ten nucleotides and the last two, and filtered for quality (Q30), length (>200), and artifacts (duplications, Ns) with FASTQ Quality Filter (hannonlab.cshl.edu/fastx_toolkit/, last accessed February 11, 2019) and Prinseq-lite 0.20.4 (Schmieder and Edwards 2011). The genome of CVB3 Nancy strain (GenBank accession JX312064) was used as reference for mapping and single-nucleotide polymorphism calling with ViVan 0.43 (Isakov et al. 2015).

### Site-Directed Mutagenesis, In Vitro Transcription, and Transfection

Self-complementary pairs of primers containing the desired mutation were designed and 150 ng of the CVB3 infectious clone were used as template for amplification with Phusion High-fidelity DNA Polymerase (ThermoScientific) under the following thermal profile: initial denaturation at 98 °C for 1 min, 25 cycles of 98 °C for 1 min, 1 min at 65 °C, and 72 °C for 6 min, followed by 10 min of final extension at 72 °C. Products were digested with *Dpn*I (ThermoScientific) at 37 °C for 2 h to remove the methylated template. Then, these reactions were transformed in NZY5α competent cells (Nzytech) and the resulting colonies were tested by Sanger sequencing for the presence of the mutation, amplified, and used for plasmid extraction by the miniprep method. Plasmids were linearized with *Sal*I (ThermoScientific) and transcribed in vitro using the TranscriptAid T7 High Yield Transcription Kit (ThermoScientific). RNA was purified by LiCl precipitation following the manufacturer’s protocol and used to transfect 400 µl of HeLa-H1 cells (10^6^ cells/ml) by electroporation in a BIO-RAD GenePulser Xcell (240 V, 950 µF in 4-mm cuvettes).

### Viral Spread and Competition Assays

To follow infection progression, cells were inoculated with CVB3-mCherry N63H or WT at 0.001 PFU/cell of S fraction. For competition assays, a 1:1 mixture of eGFP- and mCherry-encoding viruses was used. Automated real-time quantitative fluorescence microscopy was used to track the spread of each virus in an IncuCyte S3 Live-Cell Analysis System (Essen BioScience) placed inside a standard direct-heat cell culture incubator. Images were captured regularly in the red and/or green channel with a 4× objective. Background correction by the Top-Hat method and binarization for quantitation of the mCherry- or eGFP-positive area were performed using IncuCyte proprietary software.

### Cytotoxicity Assays

IncuCyte Cytotox Red Reagent for counting dead cells (Essen Bioscience) was added to infection media and images were captured and analyzed by automated real-time quantitative fluorescence microscopy.

## Supplementary Material

Supplementary data are available at *Molecular Biology and Evolution* online.

## Supplementary Material

msaa208_Supplementary_Data
