# Supplementary material for: Experimental Evolution Reveals a Genetic Basis for Membrane-Associated Virus Release
Source: Mol Biol Evol. 2020 Aug 18;38(2):358–67. doi: 10.1093/molbev/msaa208 (PMC7826177; doi:10.1093/molbev/msaa208)
Supplement: msaa208_Supplementary_Data [file msaa208_supplementary_data.zip › msaa208-suppl_data/Fig_S1.pdf]

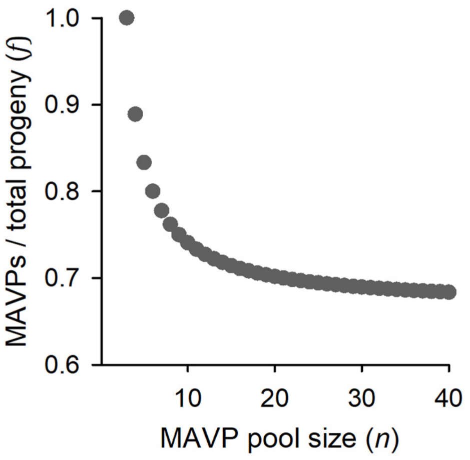

Figure S1. Model linking the number of infectious particles contained in MAVP pools (*n*) with the fraction of MAVPs to total infectious particles (*f*). As shown in the main text, $f = n/(n-1)(1 - T/T')$, where T'/T is the effect of membrane disruption on overall titer ($T'/T \approx$ 3.2 according to our data). If all infectious units were made of MAVPs (f= 1), then $T'/T = n$. Also, if MAVP pools contained a very large (infinite) number of infectious particles, $f = 1 - T/T'$ (here, 0.69). This shows that, according to our data, most infectious particles released by the WT virus were in the form of MAVPs, regardless of MAVP pool size.
