# Supplementary material for: Experimental Evolution Reveals a Genetic Basis for Membrane-Associated Virus Release
Source: Mol Biol Evol. 2020 Aug 18;38(2):358–67. doi: 10.1093/molbev/msaa208 (PMC7826177; doi:10.1093/molbev/msaa208)
Supplement: msaa208_Supplementary_Data [file msaa208_supplementary_data.zip › msaa208-suppl_data/Table_S1.pdf]

Table S1. List of all mutations found at >5% in at least one line.

Mutation	Protein	Residue change	Founder	*s1*	*s2*	*p1*	*p2*	*bp1*	*bp2*	*p*1*	*p*2*	*bp*1*	*bp*2*
A26G	5UTR	-	2.1	1.1	5.2	2.3	2.7	0.3	3.5	4.5	0.4	2.2	1.6
C27U		-	3.0	13.5	5.6	6.0	8.6	3.7	5.6	7.5	4.4	6.9	4.1
C28U		-	4.8	13.8	11.1	21.6	20.8	18.3	19.6	27.7	21.6	23.1	15.2
C29U		-	2.8	9.7	5.5	14.5	11.3	12.0	9.5	10.4	21.9	16.1	14.4
A30U		-	7.1	2.0	0.4	0.3	2.3	0.8	1.0	1.8	1.2	1.2	0.4
A30G		-	8.1	5.7	11.2	11.3	16.3	19.5	20	16.0	20	14.3	18.1
C31U		-	4.6	5.1	4.7	23.4	16.3	26.9	20	8.6	18.5	17.7	26.1
A32G		-	4.0	5.7	12.9	13.6	12.8	10.6	12.2	15.6	6.4	12.6	15.1
C90U		-	0	0.1	0	4.1	2.7	5.9	5.0	0.8	1.5	4.6	3.3
A139G		-	0	0	0.1	0	5.2	0	0	0	0.1	0	0
A266U		-	0	0	0	0	8.1	0	0	0	0	2.7	0
A312G		-	0	20.6	0	0	0	0	0	0	0	0	0.6
G499U		-	0	0	0	0.5	0.4	1.3	0.1	6.4	0.6	0.4	10.6
C655U		-	0	0	0	0	0	0	0	10.3	0	0	0
C719U		-	0	0.1	0.1	0	5.3	0	0	0	0	0	0
G749A	VP4	A3T	0	54.4	0	0	0	0	0	0	0	0	0.9
U796A		N18K	0	8.9	63.8	0	0.2	0	4.5	0	0.1	0	0
U808A		N22K	0	0	0	0	0	0	0.1	0	6.7	0	0
U813C		I24T	0	0.2	0.8	77.6	57.1	64.4	44.7	27.4	33.3	55.7	46.6
A958U	VP2	-	0	0	11.3	0	0	0	0	0	0	0	0
U992A		S15T	0	5.6	10.7	0	0	0	0	0	0	0	0
A1117G		-	0	0	0	0	0	0	5.9	0	0	0	0.1
G1210A		-	0	0.2	0	5.2	2.1	3.0	0.7	3.4	3.0	3.5	2.9
U1294C		-	0	0.1	0.1	0	6.3	0	0.6	0	0	0	0
A1354U		-	0	0	0	62.0	26.4	46.4	32.2	22.8	48.6	37.5	30.6
G1577A		V210I	0	8.1	0.9	0	0	0	0	0	0	0	0
G1622A		V225I	0	5.7	20	0	0	0	0	0	0	0	0
G1687A		-	0.1	0.1	0.4	0	0	0	0	0	5.6	0	0.2
G1708A		-	0	0	0	9.4	3.7	3.3	7.6	5.1	20.7	11.9	0
A1720G		-	0	0.7	0.1	0	5.1	0	0	0.5	0.1	0	0.8
G1842U	VP3	R35M	0	0	0	0	5.9	0	0	0	0	0	0
A1925C		N63H	0.9	98.9	97.7	0.1	0.1	1.2	1.1	0	0.1	0.2	2.0
A1939G		-	0	0.1	0.1	8.4	2.9	3.8	7.2	4.3	15.9	10.9	0
U2175C		V146A	0	0	0	0.9	6.1	0.1	0.1	16.0	4.4	0.2	0.1
G2344A		-	0	0.1	0.1	54.0	0.1	0.1	0.3	0.1	0.1	0.2	0.1
G2569A	VP1	-	0.1	0.1	19.8	0.5	0.2	0.2	0.4	0.2	0.2	0.2	0.2
C2641U		-	0	0	0	0	0.1	0.1	7.8	0.1	0	0.1	0.1
A2690G		K80E	0	0	0.1	10.8	1.3	0	0.1	3.9	18.7	0	0.1
U2696A		S82T	0	20	0	0	0	0	0	0	0	0	0
G2900A		V150I	0.1	19.9	30.1	0.1	0	12.9	0.6	0.1	0	0.1	0.1
A3005G		I185V	0	0	0	0.9	8.8	1.3	0.2	28.6	1.4	0.1	0
A3140G		K230E	0	0	0	1.0	0.4	0.1	0	1.3	24.6	0.1	0
A3196G		-	0	0	0	0.1	5.5	0.1	0	0.1	0	0.1	0

A3221C		K257Q	0.1	0	0	80.6	72.8	2.1	0.8	42.5	49.8	0.5	1.4
A3222U		K257M	0.1	0.2	0	18.9	26.5	84.5	96.7	56.9	49.4	99.3	98.2
A3231G		N260S	0	0	0	0.2	0.3	0	0	0.2	6.0	0	0
U3302C	2A	F3L	0	0.1	0.1	0.2	0	0	7.2	0.1	0.1	0	0.1
U3303C		F3S	0	57.5	0	0	0	0	0	0.1	0.1	0	0
U3352C		-	0	0.1	0	53.8	0.1	0.1	0	0.1	0.1	0.3	0
A3409G		-	0	0	0	1.8	4.6	13.6	16.9	11.9	4.7	16.4	13.1
C3519U		S75L	0	0.4	28.8	0	0.1	0.1	0.1	0.1	0.1	0	0
C3622U		-	0	0.4	0	0	0.1	0.1	0.1	0.1	6.1	0.1	0.1
G3810A	2B	C22Y	0	0	17.0	0.9	1.1	6.5	0.7	0.2	0.2	0.3	0.6
A3822G		N26S	0	0	0	5.4	0	0.1	0.1	0.1	0	0.1	0.2
G3913A		-		0	0.1	0	8.2	0	0	0	0	0	0.1
A3955G		-	0	0.4	30.8	0	0.1	0	0.1	0	0.1	0.1	0
A4123G	2C	-	0	0	18.1	0.2	0.2	0	0.1	0.1	0.1	0.2	0.2
U4711C		-	0	0.1	0.4	0.4	0.2	7.9	0.2	0.1	0.1	0.3	0.1
A4717U		-	0.6	2.5	1.8	3.5	1.9	1.9	2.8	2.6	10.2	3.2	2.2
C4735U		-	0.1	0.1	0.1	0.1	0.1	0.3	0.2	0.1	16.6	0.4	0.4
U4909C		-	0	0.1	0.2	0.2	0.1	0.2	0.1	9.1	0.4	0.2	0.2
C5044U	3A	-	0.1	0.1	5.8	0.2	0.1	0.1	0.1	0.2	0.1	0.2	0.1
A5093G		I22V	0.1	0.7	0.3	7.9	2.8	3.9	3.2	0.3	1.4	0.7	5.3
C5198U		H57Y	0	20.4	0.4	1.8	4.6	0.7	2.5	1.6	0.9	1.1	1.4
G5205C		S59T	0	0	0	0.4	0	7.9	0.3	0	0	0	0.1
A5224G		-	0.1	0.1	0.1	54.6	0.2	0.2	0.3	0.3	0.1	0.2	0.3
G5312A	3B	V6M	0	0.1	0.3	0.3	0.2	7.6	0.3	0.1	0.2	0.4	0.6
A5356G		-	0.1	56.6	13.4	0.1	0.5	0.2	0.1	0.2	0.1	0.1	0.1
C5782U	3C	-	0	0	0.1	0	7.2	0.4	0.1	0	0	0	0.1
U5952A	3D	F14Y	0	0	18.7	0	0	0	0.3	0	0	0	0
A6115U		-	0	0	0	52.3	0	0	0	0	0	0	0
A6142G		-	0	0	0	0.1	0.1	0	0.6	8.9	0.1	0.2	9.7
G6202A		-	0	0	0	0.1	8.6	0.2	0	0.1	0	0	0
U6271C		-	0	0	0.1	9.4	0.2	0.1	0.1	0	0.1	0.1	0.1
U6405C		I165T	0	0.2	0.1	0.2	0.5	0.3	0.8	11.3	0.6	0.4	9.9
C6712U		-	0	0.1	19.2	0.1	0.1	0.1	0.1	0.1	0.2	0.3	0.1
A6905C		I332L	0	0	0.1	0	0	0	0	0.2	19.6	0	0.1
A7019G		T370A	0	0.1	6.5	0.4	0.2	0.2	1.2	0.6	0.4	0.3	0.2
C7066U		-	0	0	0.1	13.1	0.2	0.1	0.1	0.1	0.1	0.2	0.1
C7258U		-	0.1	1.6	0.2	0.8	6.2	0.4	0.4	0.3	0.4	0.4	0.4
C7294U		-	0.2	0.7	1.8	0.9	1.8	3.1	3.5	12.8	1.8	4.0	14.6
C7357U	3UTR	-	0.1	0.4	10.4	1.3	0.4	1.5	0.5	0.7	1.0	1.0	1.0
